# Pulmonary squamous cell carcinoma with duodenal metastasis: A case report of immunological hyperprogression

**DOI:** 10.1097/MD.0000000000043425

**Published:** 2025-07-25

**Authors:** Qingwang Hua, Zhenyun Ye, Suyue Liu

**Affiliations:** a Department of Thoracic Surgery, Ningbo No. 2 Hospital, Ningbo, Zhejiang Province, China.

**Keywords:** gastrointestinal metastasis, hyperprogression, lung cancer

## Abstract

Immune checkpoint inhibitors have significantly improved the treatment of non-small cell lung cancer by enhancing antitumor immune responses. However, not all patients achieve favorable outcomes. Immune hyperprogression is not uncommon in current adjuvant therapy, but the occurrence of metastatic tumors in the form of hyperprogression when the primary tumor is well controlled has not been reported. This case presents a patient who developed immune hyperprogression (duodenal metastasis) during the immune maintenance phase after surgery. This case underscores the potential for ectopic metastases and hyperprogression during immunotherapy. Clinicians are reminded of the importance of cautious immunotherapy, early identification of HPD, and personalized follow-up in the management of immunotherapy for non-small cell lung cancer.

## 1. Introduction

Non-small cell lung cancer (NSCLC) commonly metastasizes to the brain, bones, liver, adrenal glands, and lungs, but gastrointestinal metastases are exceedingly rare, particularly in the form of immune hyperprogression (HPD), which has not been previously reported. Here, we present a case of duodenal metastasis from NSCLC. After imaging and pathological diagnosis of metastatic lesions, immune HPD was considered.

## 2. Case presentation

A 69-year-old male patient (BMI: 20.28 kg/m^2^) was admitted for treatment due to “a right upper lung mass with mediastinal lymphadenopathy for 1 week.” The PET-CT staging revealed cT3N2M0, Stage IIIb (eighth UICC). Bronchoscopy indicated a new mass (65 × 52 × 31 mm) at the right upper lung bronchial opening, and biopsy pathology confirmed poorly differentiated squamous cell carcinoma. Immunohistochemistry showed CK5/6(+), P40(+), and Ki-67(+50%), CK7(−), Napsin A(−), TTF(−), Syn(−), CgA(−). After excluding immuno-chemotherapy contraindications, the patient received a neo-adjuvant regimen consisting of DP + PD-1 (docetaxel 100 mg day 1, carboplatin 450 mg day 1, and toripalimab 240 mg day 1, every 3 weeks). After 3 cycles of treatment, the tumor significantly shrank (35 × 24 × 19 mm), and a cavity formed, achieving partial remission. One month after the third chemoimmunotherapy, the patient underwent single-port thoracoscopic right upper lobectomy and mediastinal lymph node dissection. Postoperative pathological staging revealed ypT1N0M0. The patient had an uneventful recovery, and after the end of chemoimmunotherapy, continued maintenance immunotherapy with a single agent. After the 2-month postoperative follow-up indicated good recovery of the lung with no evidence of tumor recurrence or metastasis. Ultrasound examination of the upper abdomen was normal, and the patient had no discomfort such as choking or vomiting while eating.

**Figure 1.**
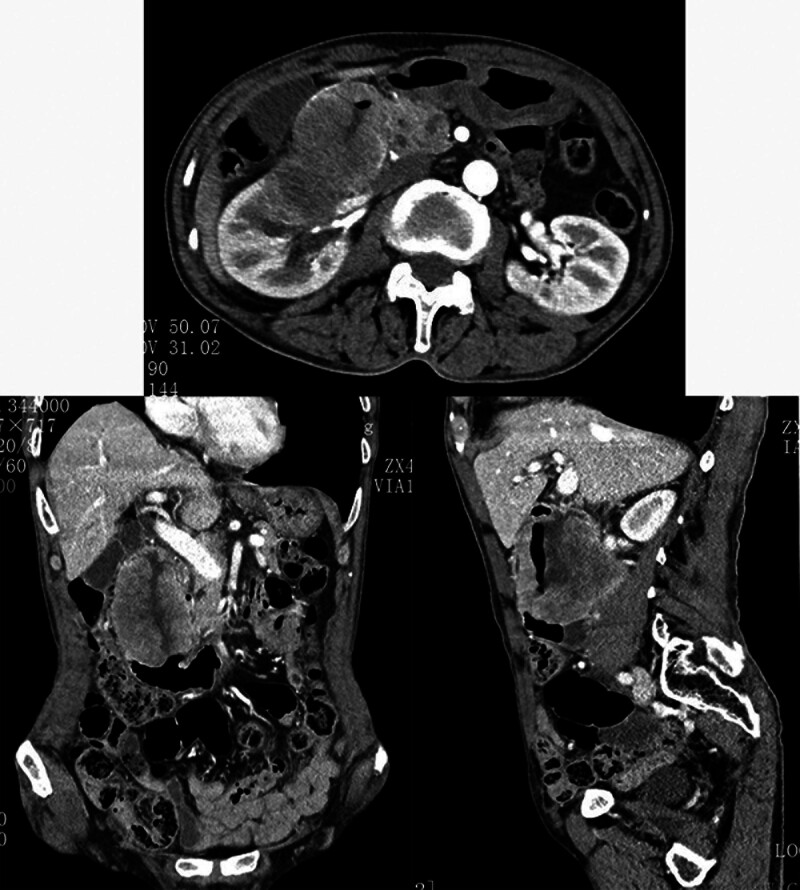
Pulmonary squamous cell carcinoma metastasizes to the duodenum and invades surrounding tissues.

**Figure 2.**
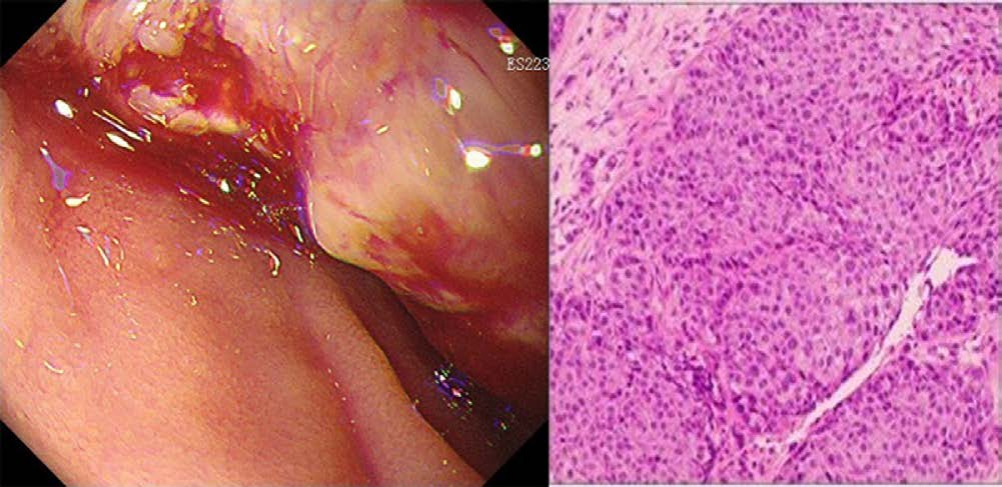
Gastroscopy schematic diagram and biopsy pathology.

However, only 1.5 months passed (the 4th month after surgery), the patient suddenly presented with hematemesis and melena. A thorough examination revealed a large retroperitoneal mass (96 × 55 × 81 mm) that had invaded and infiltrated the surrounding tissues (Fig. 1). The gastroscopy indicated a new lesion in the duodenum and biopsy pathology confirmed squamous cell carcinoma (Fig. 2), suspected to be a metastasis from the primary pulmonary tumor.

Despite symptomatic treatment including nutritional support and drug anlotinib, the patient’s symptoms did not improve. Due to persistent vomiting accompanied by upper gastrointestinal bleeding and cachexia, the patient ultimately succumbed to the disease after 1.5 months.

## 3. Discussion

Although immunotherapy has become an important modality in the treatment of NSCLC, particularly squamous cell carcinoma, this case highlights that some patients may experience immune escape or accelerated tumor growth, leading to HPD and consequently a worse prognosis. Metastatic manifestations are not uncommon in the treatment of NSCLC; however, gastrointestinal metastases resulting from immune HPD have not been reported.^[1]^

Extensive clinical data indicate that HPD typically occurs within the first 2 months of anti-PD-1/PD-L1 therapy,^[2]^ with rare cases reported after combination therapy or multiple cycles, especially during the immune maintenance phase. The definition of HPD remains contentious,^[3,4]^ and its underlying mechanisms are yet to be elucidated.^[5]^ The diagnosis in this case was substantiated by progressive clinical symptoms and pathological confirmation of the metastatic lesion. Compared to pseudoprogression,^[6]^ the patients with immune HPD exhibit poorer prognoses.^[7]^ Despite immediate discontinuation of immunotherapy and initiation of corticosteroid treatment, the patient’s symptoms did not improve and continued to worsen. Targeted therapy was not an option due to the squamous histology, and rescue treatment with anlotinib was also ineffective in shrinking and controlling tumors.^[5]^ The patient’s PS score was 3, so he did not receive any subsequent chemotherapy in the later period.^[8]^

The lesson of this case makes us reflect on the double-edged sword effect of immunotherapy: for patients receiving postoperative immunotherapy maintenance therapy, on the 1 hand, the DFS is significantly improved, but on the other hand, the immune-related adverse events increase accordingly.^[9]^ Patients undergoing immunotherapy require close monitoring, particularly those with poor baseline health or heavy tumor burden. The potential side effects and risk of HPD after immunotherapy should be thoroughly considered.^[10]^ In this case, duodenal metastasis of NSCLC, as an ectopic metastatic form of pulmonary squamous cell carcinoma, is rare in the treatment of lung cancer. Its early symptoms are not significant and can easily be masked by chemotherapy and immune-related adverse effects, leading to potential oversight. We also reflected on whether to reduce the duration of immunotherapy maintenance for patients who underwent R0 resection after neo-adjuvant surgery to balance efficacy and immune-related adverse events as such patients are not uncommon.

This case highlights the possibility of ectopic tumor metastasis development due to immunotherapy-induced HPD, reminding us that follow-up should not be limited to common metastatic sites of lung cancer. The current follow-up criteria mainly focus on chest imaging and examination of common metastatic sites, but for complex cases after immunotherapy, a more comprehensive evaluation should be considered. A personalized follow-up model should be developed based on the patient’s clinical characteristics, treatment response, prognostic risk, etc, and special attention should be paid to the protection of vital organs to improve individual survival rate.^[9]^

In conclusion, this case highlights the significant impact of tumor metastasis and HPD after immunotherapy, emphasizing the importance of cautious immunotherapy and early identification of HPD. In clinical practice, especially with the increasing use of immunotherapy, the individualized and precise approach to treatment decision-making and follow-up planning is crucial.

## Author contributions

**Conceptualization:** Qingwang Hua.

**Data curation:** Zhenyun Ye.

**Supervision:** Suyue Liu.

**Writing – original draft:** Qingwang Hua.

**Writing – review & editing:** Suyue Liu.
